# Prevalence of endodontic infection in patients with Crohn´s disease and ulcerative colitis

**DOI:** 10.4317/medoral.24135

**Published:** 2020-08-27

**Authors:** Manuel Poyato-Borrego, Juan J. Segura-Egea, Jenifer Martín-González, Mari Carmen Jiménez-Sánchez, Daniel Cabanillas-Balsera, Victoria Areal-Quecuty, Juan J. Segura-Sampedro

**Affiliations:** 1 MD, PhD. Clinical Unit of Infectious Diseases, Microbiology and Preventive Medicine. Infectious Diseases Research Group, Institute of Biomedicine of Sevilla (IBiS). University of Sevilla/CSIC/University Hospital Virgen del Rocio. Sevilla, Spain; 2 MD, PhD, DDS, Professor. Department of Stomatology, Section of Endodontics, School of Dentistry, University of Sevilla, Spain; 3 DDS, PhD, Associate Professor. Department of Stomatology, Section of Endodontics, School of Dentistry, University of Spain; 4 DDS, Doctoral fellow. Department of Stomatology, Section of Endodontics, School of Dentistry, University of Sevilla, Spain; 5 MD, PhD, Associate Professor. General and Digestive Surgery Unit. Son Espases University Hospital. School of Medicine, University of Balearic Islands. Palma de Mallorca, Spain

## Abstract

**Background:**

Previous studies have linked apical periodontitis (AP) to inflammatory bowel disease (IBD). The aim of this study was to compare the prevalence of AP and root canal treatment (RCT) in patients with ulcerative colitis (UC) and Crohn´s disease (CD).

**Material and Methods:**

A cross-sectional study, including 28 patients with Crohn´s disease and 26 with ulcerative colitis, was conducted. AP was diagnosed as radiolucent periapical lesions (RPLs), using the periapical index score (PAI). Student’s t test, 2 test and multivariate logistic regression were used in the statistical analysis.

**Results:**

Multivariate logistic regression run with age, gender, number of teeth, number of RFT, periodontal disease and the type of IBD as covariates, taking as dependent variable and outcome “periapical status” (0 = no tooth with RPL; 1 = at least one tooth with RPL), showed that both UC and CD patients had the prevalence apical periodontitis (OR = 1.03; C.I. 95% = 0.25 – 4.31; *p* = 0.97). The multivariate analysis, including all the above covariates, shows that both in UC and CD patients the prevalence of RCT was similar (OR = 0.76; C.I. 95% = 0.17 – 7.31; *p* = 0.73). Periapical status was significantly associated with endodontic status (OR = 42.72; C.I. 95% = 3.87 – 472.15; *p* = 0.002), regardless of IBD type.

**Conclusions:**

The results of the present study show similar frequency of AP and RFT in both UC and CD patients. The type of IBD does not appear to affect the prevalence of radiographically detectable periapical lesions or the prevalence of root canal treatment.

** Key words:**Apical periodontitis, Crohn’s disease, inflammatory bowel disease, toot canal treatment, ulcerative colitis.

## Introduction

Inflammatory Bowel Diseases (IBD) include Crohn's disease (CD) and ulcerative colitis (UC), two recurrent and chronic inflammatory processes of the gastrointestinal tract characterized by diffuse inflammation of the intestinal mucosa. CD affect, in most cases, terminal ileum, but can affect any segment of the gastrointestinal tract, from the mouth to the anus. On the other hand, UC affects the large bowel, being the distal colon the most affected region (1). Oral manifestations, including angular cheilitis, mucosal edema, granulomatous gingivitis, and linear ulceration, affect 4% - 16% of IBD patients (2).

On the other hand, when caries progression causes irreversible pulpitis and pulp necrosis, root canal poly-microbial antigenic content escapes through the apical foramen, invading the periapical tissues. Then, an inflammatory response develops, named apical periodontitis (3). Apical periodontitis (AP) has an endodontic origin, being a sequel of tooth decay. Chronic AP is characterized by inflammatory resorption of alveolar bone, evidenced radiographically as a radiolucent periapical lesion (RPL) around the apex of the affected tooth. AP is a very prevalent disease all over the world (4), being root canal treatment (RCT) the elective therapy for teeth with AP.

In the last years, an association between AP and some systemic diseases, such as diabetes mellitus (5) and cardiovascular disease (6), has been found. This association would be based on the presence of a pro-inflammatory status, as well as risk factors common to both AP and the systemic disease (7). Furthermore, previous studies have also found association between the prevalence of AP and IBD, with higher prevalence of AP (8,9) and larger periapical lesions (8) in IBD patients, compared to healthy control subjects. These studies included both UC and CD patients in the sample. The aim of this cross-sectional study was to compare the prevalence of AP, assessed as RPL, and the prevalence of RCT, in patients with UC and CD. The null hypothesis was that there are no significant differences in the prevalence of endodontic variables between UC and CD patients.

## Material and Methods

- Patients’ selection

Participants were recruited among patients with IBD receiving treatment at the San Juan de Dios University Hospital (Sevilla, Spain) between the years 2017 and 2019. Patients diagnosed as CD and UC according to the international investigational protocols and following the Montreal classification of IBD (10), were consecutively asked to volunteer until the minimum sample size was widely exceeded. Inclusion criteria were as follows: patients older than 18 years, having at least 8 remaining teeth, who agreed a radiological examination. Exclusion criteria encompassed patients younger than 18 years old, having less than 8 remaining teeth, or who did not agree a radiological examination. Only two patients refused to participate. A total of 54 patients, 31 men and 23 women (43.1 ± 14.0 years) that agreed and met the inclusion/exclusion criteria were included in the study. The sample included 28 patients with Crohn's disease and 26 with ulcerative colitis (Table 1).

- Radiographic examination

Radiographic periapical status was diagnosed on the basis of examination of digital panoramic radiographs of the jaws. Two trained radiographic technicians, with over ten years of experience, took the panoramic radiographs using a digital ortho-pantomograph machine (Promax®, Planmeca, class 1, type B, 80 KHz, Planmeca, Helsinki, Finland).

- Radiographic evaluation

The periapical status was assessed using the “Periapical Index” (PAI) score (11), considering sign of periapical pathology a score greater than 2 (PAI ≥ 3). For multi-rooted teeth, the worst score of all roots was taken to represent the PAI score. Teeth filled with a radiopaque material in the root canal(s) were categorized as root-filled teeth.

For each patient, the following data were recorded on a structured form: (a) number of teeth present; (b) number and location of teeth having identifiable radiolucent periapical lesions, (c) number and location of root-filled teeth, and (d) number and location of root-filled teeth having identifiable radiolucent periapical lesions.

- Observers’ calibration

Two blinded observers, with extensive clinical experience in endodontics, assessed the periapical status in radiographs. Previously, the observers had participated in a calibration course for PAI system, consisting of 100 radiographic images of teeth, some root-filled and some not, kindly provided by Dr. Ørstavik. The observers assigned one of the PAI scores to each tooth, using visual references for the five categories within the scale (11).

**Table 1:**
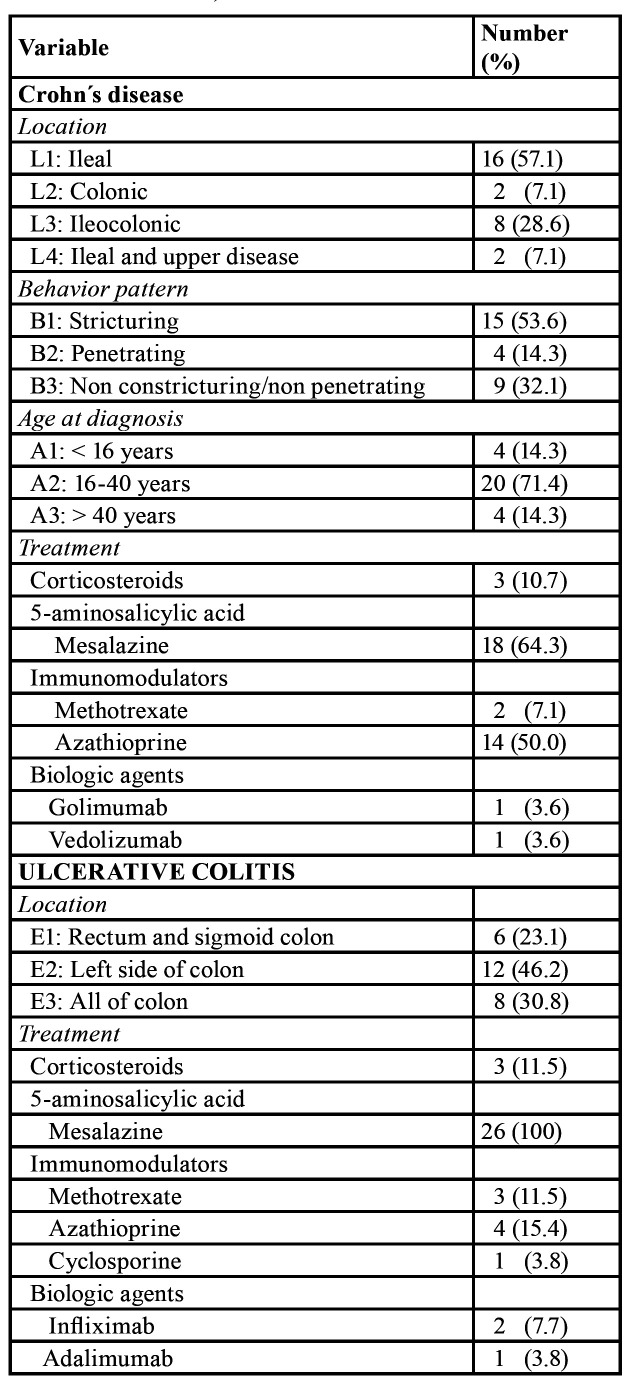
Patients with Crohn's disease (n = 28): location and behavior pattern, according to the Montreal classification, and treatment. Patients with ulcerative colitis (n = 26): location according to the Montreal classification, and treatment.

Then, the results were compared to a “gold standard atlas”, also provided by Dr. Ørstavik. Cohen Kappa was calculated (0.78 – 0.85). Intra-observer reproducibility was also evaluated for each examiner scoring the radiographs of 20 patients, randomly selected. After recalibration in the PAI system, these first results were compared with those of a second evaluation of the same 20 patients carried out one month later. The intra-observer agreement test on PAI scores on the 20 patients produced a Cohen’s Kappa ranging 0.83 - 0.94. For inter-observers variability the Cohen’s Kappa ranged 0.75 - 0.84. The consensus radiographic standard was the simultaneous interpretation by the two examiners of the panoramic radiograph of each patient.

- Statistical analysis

The prevalence of AP and RCT was evaluated on the total number of individuals and the total number of teeth. The minimal sample size (n = 49) was calculated using the sample size calculator software of the National Center for Advancing Translational Sciences (NIH, UK) (http://www.sample-size.net/sample-size-proportions/) for the comparison of proportions in two independent samples, with continuity correction. They were taken into account a two-sided significance level of 5% ( = 0.05, Z = 1.960), and 80% power ( = 0.20, Z = 0.842) to detect a hypothesized difference between the proportion of the two types of IBD of 30 points.

Raw data were entered into Excel (Microsoft Corporation, Redmond, WA). All analyses were done in an SPSS environment (Version 11; SPSS, Inc, Chicago, IL). The Student t test, 2 test, and logistic regression analysis were used to determine the significance of differences between groups. Data are reported as mean ± standard deviation. According to the established significance level, a *p* value ≤ 0.05 was considered statistically significant.

## Results

The distribution of analysed variables in the two groups is shown in Table 2. There was no significant difference in age, gender or BMI between patients with UC and CD. The average number of teeth per patient was 25.3 ± 4.3 and 24.5 ± 3.4 teeth in UC and CD patients, respectively (*p* > 0.05). Patients with UC showed similar average number of teeth with AP (0.5 ± 0.8) as patients with CD (0.5 ± 0.9) (*p* > 0.05). There were also no significant differences between the two types of IBD in the number of RFT or in the number of RFT with AP. The prevalences of periodontal disease and smoking habits were also similar in both UC and CD patients (*p* > 0.05).

Taking the patient as a reference (Table 3), in the CD group 10 patients (35.7%) had at least one tooth with radiolucent periapical lesion (RPL), whereas in the UC it was presented 9 subjects (34.6%) (*p* > 0.05). There were no differences between both groups in the number of patients with one or more RFT (*p* = 0.60) or in the number of patients with one or more RFT with RPL (*p* = 0.43).

To further investigate the variables influencing periapical status, multivariate logistic regression was run with age, gender, number of teeth, number of RFT, periodontal disease and the type of IBD, taking as dependent variable and outcome “periapical status” (0 = no tooth with RPL; 1 = at least one tooth with RPL) (Table 4).

**Table 2:**
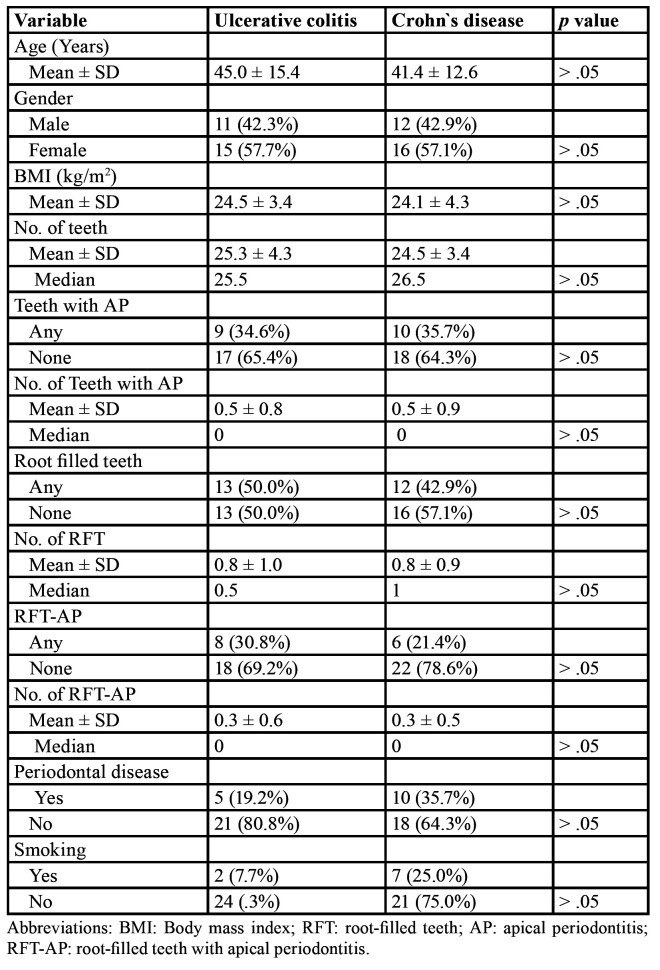
Distribution of analyzed variables among patients with ulcerative colitis (n =26) and Crohn´s disease (n = 28).

**Table 3:**
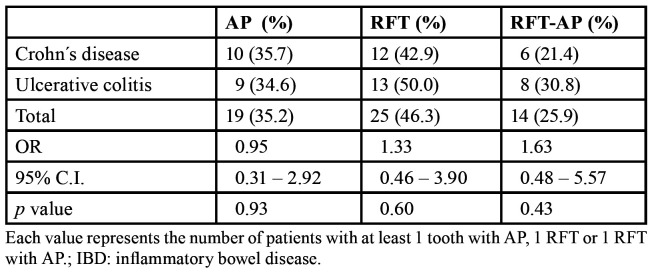
Estimation of odds ratio (OR) values, and their 95% confidence interval (C.I.), using 2 test, for the association between the prevalence of apical periodontitis (AP), root-filled teeth (RFT), and root-filled teeth with apical periodontitis (RFT-AP) in patients with IBD (Crohn´s disease, n = 28; ulcerative colitis, n = 26).

**Table 4:**
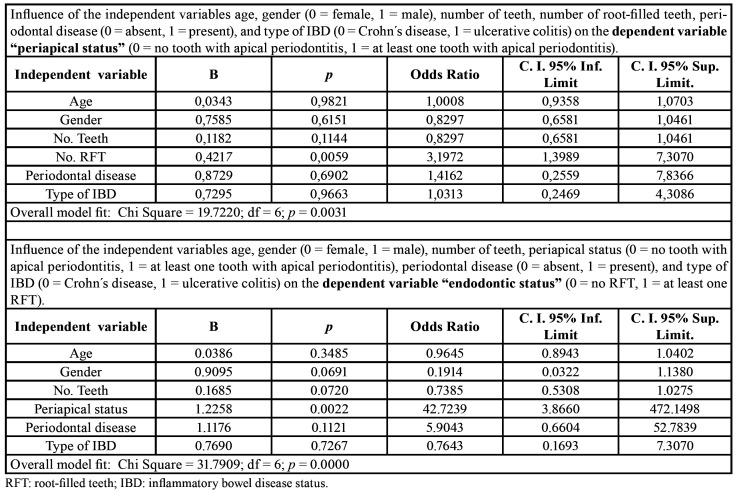
Multivariate logistic regression analysis.

In the multivariate analysis, including all the above factors as covariates, the type of IBD (OR = 1.03; C.I. 95% = 0.25 – 4.31; *p* = 0.97) was not significant, indicating that both UC and CD patients had the same likelihood of having apical periodontitis. The only variable that was significantly associated with the periapical status was the number of RFT (OR = 3.20; C.I. 95% = 1.40 – 7.31; *p* = 0.006).

Finally, to analyze the variables influencing the endodontic status, multivariate logistic regression was run with age, gender, number of teeth, periapical status, periodontal disease and the type of IBD, taking as dependent variable and outcome “endodontic status” (0 = no RFT; 1 = at least one RFT) (Table 4). In the multivariate analysis, including all the above covariates, the type of IBD (OR = 0.76; C.I. 95% = 0.17 – 7.31; *p* = 0.73) was not significant, indicating that the likelihood of having RFT was the same both in UC and CD patients. Periapical status was significantly associated with endodontic status (OR = 42.72; C.I. 95% = 3.87 – 472.15; *p* = 0.002).

## Discussion

This cross-sectional study aimed to investigate the prevalence of AP and RCT in patients with UC and CD. The null hypothesis tested, i.e. there are no significant differences in the prevalence of endodontic variables between UC and CD patients, has been confirmed. No significant differences in periapical status or endodontic status have been found between patients with UC and patients with CD.

Patients were recruited by means of the same system used in previous studies (5,8,9). Both CD and UC were diagnosed according to the current criteria (10). A relatively low percentage of patients with ileocolonic disease has been found, while, on the contrary, the percentage of patients with isolated ileal disease has been relatively high. This is in accordance with previous studies conducted in Spain (9,12).

The two compared samples of patients with UC and CD had a similar age, gender, and BMI distribution. They also showed no significant differences in the number of teeth and the number of RFT, two variables significantly associated to radiographically diagnosed periapical lesions (13). The percentages of smokers and periodontal patients were also similar in both types of IBD. The types of medical treatments followed by patients, i.e. the use of corticosteroids, 5-aminosalicylic acid, immunomodulators, and biological agents, were in accordance with current recommendations for the treatment of IBD (14).

Periapical status has been evaluated using the ‘periapical index’ (PAI) (11), which assesses the prevalence of AP through the presence of RPL. This scoring system has been widely used in epidemiological studies (9).

Results of many cross-sectional studies suggest an association between AP and systemic diseases (15), such as diabetes mellitus (16), cardiovascular diseases (6), tobacco smoking (17), and others (15). The results of these studies only demonstrate statistical association, but the existence of a causal relationship cannot be concluded from them (6,18). Nevertheless, these findings have promoted the attention to the oral health of these patients, at the same time that have led to investigate possible associations of other diseases with periapical infection.

One of the diseases whose relationship with endodontic pathology has been recently investigated has been IBD, including UC and CD (8,9,19). The etiology and pathogenesis of IBD depend on genetic and environmental factors. An inadequate immune response of the intestinal mucosa against the luminal antigens in a host with genetic susceptibility, would result in an imbalance of pro-inflammatory and anti-inflammatory factors, producing intestinal inflammation (20). Both IBD have common signs and symptoms: abdominal pains, poor appetite with weight loss, diarrhea, and rectal bleeding, showing active episodes and asymptomatic intervals (21).

Before its association to apical periodontitis, several studies had investigated the relationship of IBD with periodontal disease (22). The pathogenesis of both IBD and periodontal disease involves interplay between bacteria and the host immune-inflammatory response, greatly influenced by genetic and environmental factors (23). IBD patients show significantly higher prevalence of periodontal disease, as well as worse oral health, compared to healthy control patients (24). The consumption of dental treatment among IBD patients is greater than that of control subjects, needing both CD and UC patients significantly higher number of procedures (25).

Apical periodontitis has similar etiology and pathogenesis to that of periodontal disease (15), but few studies have so far investigated the possible association between IBD and AP or RCT (8,9). These studies have found significantly higher number of teeth with AP in women with IBD, as well as significantly higher PAI index scores in IBD patients than in healthy control subjects (8). Moreover, IBD patients are almost six times more likely to have apical periodontitis than control subjects (9). Taken together, the results of studies regarding the association between IBD and periodontal disease (22,26), together with the studies on the association between IBD and apical periodontitis (8,9), suggest that IBD is a risk factor for oral infections.

All the previously mentioned studies used samples in which there were UC patients and CD patients. Therefore, it seemed interesting and justified to carry out a study comparing the prevalence of AP and RCT in the two types of IBD. The present study contributes to solving this gap. The prevalences of AP found in UC patients (35%) and in CD patients (36%) were comparable and similar to that found in previous studies(8,9). No difference was found between the two types of IBD regarding the prevalence of endodontically treated teeth. These results contrast with those obtained by Johannsen *et al*. (25), who found a greater number of restorative treatment and RCT in CD patients. This discrepancy can be explained by the large difference in sample size between both studies: only 54 patients were included in the present study, and 2085 patients with CD and 3161 with UC were included in the study by Johannsen *et al*. (25). The joint analysis of the present results, together with those of previous studies showing a higher prevalence of both AP and RCT in patients with IBD (8,9), suggests that there are common factors in both diseases that could explain this association.

Similarly, no differences were found between the two types of IBD in the number of RFT with periapical lesions. The percentage of patients with at least one RFT associated to RPLs was similar in both types of IBD (*p* > 0.05). Periapical radiolucent lesions associated to RFT are interpreted as persistent chronic AP, but can also correspond to healing lesions after RCT, especially if the time elapsed since treatment was less than 2 years. It has been shown that periapical lesions in patients with IBD taking biologic medications heal faster after RCT (19). Taking into account that in the present study only three of the UC patients were taken adalimubad or infliximab, and only two of CD patients took golimumab or vedolizumab, this factor must be ruled out.

Multivariate logistic regression analyzes demonstrated a strong significant association between the periapical status and the endodontic status, not influenced by the type of IBD. This result is consistent with numerous previous studies in which it is concluded that the number of RFT is a factor directly linked to the number of periapical lesions (4).

The biological links between endodontic infection and IBD can be various. In IBD, a dysbiosis of the gut microbiota could contribute not only to the development of intestinal disorder, but also to the extra-intestinal inflammatory oral diseases (8), such as periodontal disease and apical periodontitis (9). Conversely, periodontal and endodontic biofilms could influence the development of IBDs (27). The immune system is implicated both in endodontic infection and in both types of IBD. UC is a TH2 type immune disease, with up-regulation of IL-5, whereas CD is a TH1 type immune disease, showing high levels of interferon gamma (IFN-), IL-12, and tumor necrosis factor alpha (TNF-α) (28). Apical periodontitis is also characterized by the triggering of both type of immune responses, being the earlier onset of Th1 response and the activation of osteoclasts by nuclear factor kappa B ligand (RANKL) the responsible factors of bone destruction around the apex of the affected tooth (29). After RCT, periapical repair depend on onset of Th2 response (30). Considering that the genotype is the main determinant of each person's immune response, this could be the link between periapical infection and both types of IBD.

The results of this study should be carefully evaluated, as it has some limitations. It is worth mentioning that has not been considered important factors influencing the prevalence of AP and RCT, such as educational level, socioeconomic status, diabetes, caries, quality of coronal restorations, quality of endodontic treatment, history of trauma (13).

## Conclusions

The results of the present study show similar frequency of AP and RFT in both UC and CD patients. The type of IBD does not appear to affect the prevalence of radiographically detecTable periapical lesions or the prevalence of root canal treatment.
